# Changes in Effective Connectivity Network Patterns in Drug Abusers, Treated With Different Methods

**DOI:** 10.18869/nirp.bcn.8.4.285

**Published:** 2017

**Authors:** Arash Zare Sadeghi, Amir Homayoun Jafari, Mohammad Ali Oghabian, Hamid Reza Salighehrad, Seyed Amir Hossein Batouli, Samira Raminfard, Hamed Ekhtiari

**Affiliations:** 1. Department of Medical Physics and Biomedical Engineering, School of Medicine, Tehran University of Medical Sciences, Tehran, Iran.; 2. Department of Nouroimaging and Analysis, Imam Khomeini Hospital Complex, Tehran University of Medical Sciences, Tehran, Iran.; 3. Department of Neurosciences and Addiction Studies, School of Advanced Technologies in Medicine, Tehran University of Medical Sciences, Tehran, Iran.

**Keywords:** Dynamic causal modeling, Functional magnetic resonance imaging, Abstinent based therapy, Methadone maintenance therapy

## Abstract

**Introduction:**

Various treatment methods for drug abusers will result in different success rates. This is partly due to different neural assumptions and partly due to various rate of relapse in abusers because of different circumstances. Investigating the brain activation networks of treated subjects can reveal the hidden mechanisms of the therapeutic methods.

**Methods:**

We studied three groups of subjects: heroin abusers treated with abstinent based therapy (ABT) method, heroin abusers treated with Methadone Maintenance Therapy (MMT) method, and a control group. They were all scanned with functional magnetic resonance imaging (fMRI), using a 6-block task, where each block consisted of the rest-craving-rest-neutral sequence. Using the dynamic causal modeling (DCM) algorithm, brain effective connectivity network (caused by the drug craving stimulation) was quantified for all groups. In this regard, 4 brain areas were selected for this analysis based on previous findings: ventromedial prefrontal cortex (VMPFC), dorsolateral prefrontal cortex (DLPFC), amygdala, and ventral striatum.

**Results:**

Our results indicated that the control subjects did not show significant brain activations after craving stimulations, but the two other groups showed significant brain activations in all 4 regions. In addition, VMPFC showed higher activations in the ABT group compared to the MMT group. The effective connectivity network suggested that the control subjects did not have any direct input from drug-related cue indices, while the other two groups showed reactions to these cues. Also, VMPFC displayed an important role in ABT group. In encountering the craving pictures, MMT subjects manifest a very simple mechanism compared to other groups.

**Conclusion:**

This study revealed an activation network similar to the emotional and inhibitory control networks observed in drug abusers in previous works. The results of DCM analysis also support the regulatory role of frontal regions on bottom regions. Furthermore, this study demonstrates the different effective connectivity patterns after drug abuse treatment and in this way helps the experts in the field.

## 1. Introduction

The neurocognitive process of drug craving in chronic drug abusers has been studied before and the brain regions involved in this process are well recognized (Wilson, Sayette, & Fiez, 2004; Sutherland, McHugh, Pariyadath, & Stein, 2012; Tang, Fellows, Small, & Dagher, 2012; Yalachkov, Kaiser, Naumer, 2012). Previous studies have reported the key role of the amygdala and prefrontal cortex in the cue-induced craving process (Bechara, Damasio, Damasio, & Lee, 1999). When exposed to drug cues, the brain regions, such as ventromedial prefrontal cortex (VMPFC) (Ben-Shahar, et al. 2013), dorsolateral prefrontal cortex (DLPFC) (Wilson et al. 2004; George and Koob 2013; Hayashi, Ko, Strafella, & Dagher, 2013; Batista, Klauss, Fregni, Nitsche, & Nakamura-Palacios, 2015), ventral striatum (Naqvi and Bechara 2009), and amygdala (Bechara, Damasio, & Damasio, 2003) display activation in different drug dependents.

The associated brain regions do not act alone but work as parts of hidden networks. The recent studies have tried to find out and quantify these networks (Chase, Eickhoff, Laird, & Hogarth 2011; Sutherland et al., 2013). The existing interactions between brain regions (nodes) can be passive or active; the passive type is called functional connectivity and the active one effective connectivity. Effective connectivity follows the theory of causality (Pearl, 2009). The causality in brain networks has been studied before, but the drug craving networks have been investigated in a few studies (Ray, Haney, Hanson, Biswal, & Hanson, 2015). Based on some studies, chronic drug use can change the pattern of brain activation networks in drug dependents when exposing to drug cues (Goudriaan, de Ruiter, Van Den Brink, Oosterlaan, & Veltman 2010; Janes, et al. 2010; Ma, et al, 2011; Lu, et al., 2012; Cisler, et al., 2013; Ding and Lee 2013a; Ding and Lee 2013b; Yang, et al., 2014). Furthermore, the regulatory effect of cortex on subcortical regions has already been proven, and their interactions follow a causal network pattern (Bechara, et al., 2001).

The causal networks can be quantified using different methods. Some methods address just the existence of the networks, but some other seek deeper to find more details. Two interesting issues in these networks are first how regions affect each other and second how they affect the relation among the regions. These networks can be quantified using effective connectivity measurement methods such as Structural Equation Modeling (SEM) (McLntosh & Gonzalez-Lima 1994; Buchel, 1997; Astolfi, et al. 2004; Laird, et al., 2008), Granger causality modeling (GCM) (Roebroeck, Formisano, Goebel, 2005; Wang, Chen, Bressler, & Ding. 2007; Sato, et al., 2010), and dynamic causal modeling (DCM).

We hypothesized that the fronto-amygdalar regulation is complex and not only the prefrontal regions such as VMPFC and DLPFC have reciprocal modulatory effects on the amygdala, but also they have indirect causal effects. The differences in the effective connectivity networks were investigated between the following three groups in our study: one group included subjects with no history of drug dependence as the control group, one group included subjects who were successfully treated drug abuse with Methadone Maintenance Therapy (MMT), and the last group included subjects who were successfully treated drug abusers with Abstinence Based Therapy (ABT) method.

## 2. Methods

The Ethics Committee of Tehran University of Medical Sciences approved the study protocol and consent form. Before scanning, the imaging procedure was described for all subjects and their written informed consents were obtained. After scanning, a counseling procedure was done for each subject to check for any probable adverse effect on the subject’s mental health, after presentation of drug-related cues.

### 2.1. Participants

Three study groups, each including 20 male subjects, were scanned. One group included subjects (with at least 3 months of opiate abstinence) who were successfully treated by MMT based method; the second group (with at least 3 months of opiate abstinence) included subjects who were successfully treated by ABT based method; and the third group comprised control subjects age-matched with two other groups, who did not have any history of drug abuse. The demographic characteristics of the three groups are presented in Table 1.

**Table 1:** The demographic characteristics of the three study groups.

**ABT**	**Stats**
Age	32±2.1
Gender (male)	15
Education (year)	11.1±1.18
Abstinence duration (month)	15.6±4.1
Opium abusers	15
Heroin abusers	15
Alcohol abusers	12
Tobacco users	15
**MMT**	
Age	34.7±2.52
Gender (male)	13
Education (year)	11.2±1.7
Abstinence duration (month)	16.4±3.82
Opium abusers	13
Heroin abusers	13
Alcohol abusers	10
Tabaco users	13
**Control**	
Age	28.9±2.55
Gender (male)	16
Education (year)	13.2±1.46
Alcohol abusers	0
Tobacco users	0

### 2.2. Functional magnetic resonance imaging task

**Figure 1:**
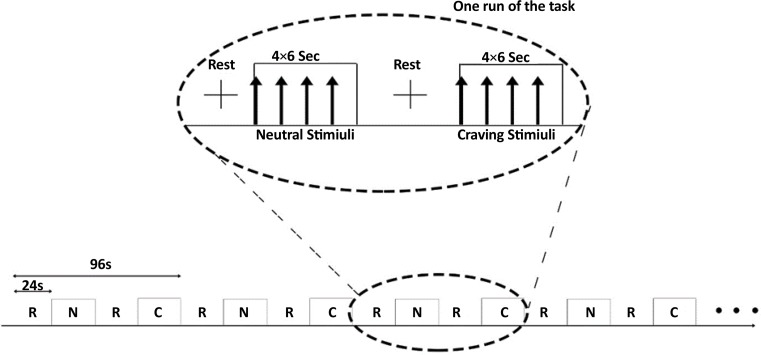
The task structure. R: represents Rest, C: represents Craving, and N: represents Neutral. There are 6 runs in the task each for 96 seconds.

The task was a block design task containing 6 consecutive runs. Each run included one rest block of 24 s length (a cross was shown), one block of 24 s length as neutral (4 images not related to heroin, each for 6 s, were shown to the subject), a second rest block, and a block of 24 s length as craving stimuli (4 images related to heroin, each for 6 s, were shown to the subject). The images (24 heroin-related and 24 neutral) were selected from International Affective Picture System (Lang, Bradley, & Cuthbert, 2005). The structure of the task is displayed in Figure 1.

### 2.3. Functional magnetic resonance imaging data acquisition

Functional images were acquired with an Avanto 1.5T scanner (Siemens, Germany) with 8 channel head coil. The T2*-weighted images were acquired with TR=3000 ms, TE=50 ms, flip angel=90°, voxel size of 3×3×3 mm^3^, and matrix size of 64×64. Each volume was composed of 36 slices which covered the whole brain in axial direction. Structural image was acquired with the following specification: T1-weighted with TR=1910, TE=3.55 ms, flip angel=30°, voxel size of 1×1×1 mm^3^, and matrix size of 256×256. The stimuli were presented using MR compatible goggles.

### 2.4. Preprocessing

FSL5 (Jenkinson, Beckmann, Behrens, Woolrich, Smith, 2012) MCFLIRT (Jenkinson & Smith 2001; Jenkinson, Bannister, Brady, & Smith, 2002) was used to correct the EPI images for the head motion. Slice timing correction was done using interleaved order, high-pass temporal filtering was done with the size of 96 s to remove the signal trend, a 3D Gaussian kernel with the size of 5 mm FWHM was used to smooth the functional images, and for group comparison the intensity normalization was done as the last part of the preprocessing step.

### 2.5. Data analysis

The purpose of this study was not to examine the between group differences with regard to regional activations, so we did only within group analyses. Using FLAME (FMRIB’s Local Analysis of Mixed Effects), we included all 4 possible contrasts; i.e., craving, neutral, craving>neutral, and neutral>craving. Based on the results, only the craving>neutral contrast supports the idea of stronger activation during watching craving cues vs. watching neutral images.

### 2.6. Time-series extraction

According to our neuroscientific hypothesis, we chose 4 regions of interests (ROIs): VMPFC, DLPFC, ventral striatum, and amygdala. These regions have been shown to be active during a drug craving task. First we made a mask for each region in MNI space, then using transformation matrices, the masks were resliced and registered to each subject’s EPI images. These matrices were calculated during registration in preprocessing step (standard2example_func.mat) and applied using the ApplyXFM tool in FSL5. The greatest eigenvariate of the voxels in each region was used as the time-series of the ROI. The extraction of eigenvariates from the time series across the voxels within each ROI was done using a singular value decomposition (SVD) method (Alter, Brown, Botstein, 2000). We used SPM12^1^ Eigenvariate Tool for achieving this purpose.

### 2.7. Dynamic causal modeling

Effective connectivity means the causal interrelation of the regions in the brain; however, this relation is in the neuronal level which cannot be measured by fMRI. Dynamic causal modeling as an established method to quantify the effective connectivity includes 4 connectivity matrices which display the strength of interconnections. The first matrix (A) contains the strength of endogenous links; these are the interrelations of regions in the absence of any input, the second matrix (B) contains the strength related to the effects of inputs on the links between regions, the third matrix (C) contains the direct strength of links of input effects on the regions and the last matrix (D) shows the strength of nonlinear links, which exerts from regions on the links connecting other regions. The equation which dominates the relation of these matrices is as follows:

$$ż=f\left(z,u,\theta \right)=Az+\left\{\sum_{j=1}^{m}u_{j}B(j)+\sum_{i=1}^{n}z_{i}D(i)\right\}z+\mathrm{Cu}$$

**Figure 2:**
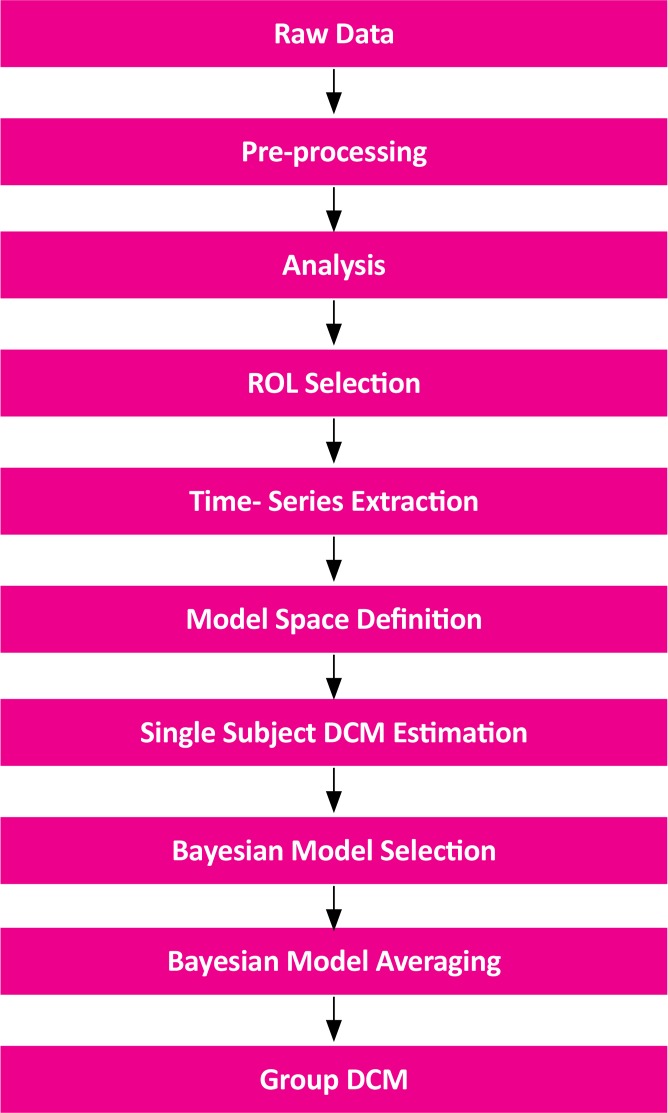
The diagram of calculating DCM.

Computing DCM for a group of subjects include some steps, which are shown in the Figure 2. Our model space contained 38 models, which reciprocally connected 4 regions; the craving input emerged to various regions; linear and nonlinear links; and self-inhibitory links. The diverse models in the model space were used to answer different neuroscientific questions. Next we estimated all models for each subject to reach the exceedance probability measure for single subject analysis and these measures were used in the Bayesian model selection (BMS) (Stephan, Penny, Daunizeau, Moran, & Friston, 2009) process to compare the models. Evidently, comparing single models does not simply provide any useful information, however, dividing the model space into families with similar features can yield the best result (Penny, et al., 2010). Thus, we divided the models into families according to their nonlinear links; separating linear and nonlinear models. Bayesian Model Averaging (BMA) was used to reach the final model. Also, we used SPM DCM12 Toolbox for computing the DCM network.

## 3. Results

### 3.1. GLM results

**Figure 3:**
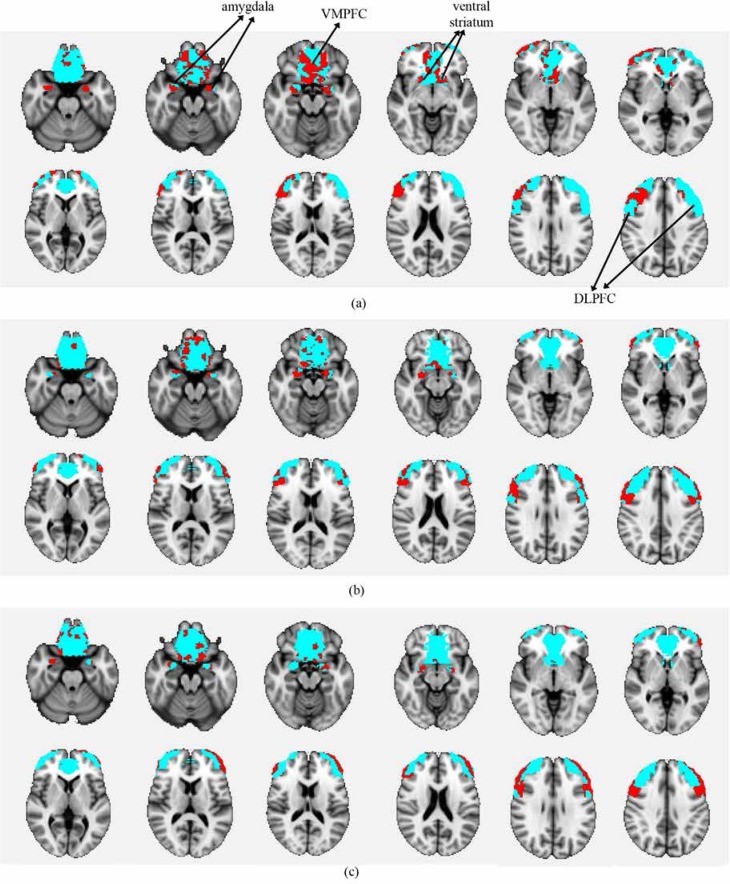
Depicting the region-based activation analysis results for three study groups; (a) ABT, (b) MMT, and (c) Control. The blue regions are the ROIs in which the activations were investigated and the red regions are the active parts during the task and specifically the craving>neutral contrast.

Statistical analysis of fMRI data of each group was done using FSL5 and the results indicated activations in all regions of interest. Figure 3 depicts the activation patterns in one of the defined contrasts (craving>neutral) and Tables 2, 3, and 4 present the group level results for all study groups (same contrast).

**Table 2:** The group level activation results in (Craving>Neutral) contrast for ABT group.

**Anatomical Regions**	**Cluster Size**	**Z-values**	**Local Maximum Co-Ordinates**		
Supracalcarine cortex	3123	4.07	0	−90	8
Lingual gyrus		3.96	2	−84	6
Lingual gyrus		3.93	−12	−76	−8
Cuneus		3.91	−10	−98	12
Retrosplenial cortex		3.66	12	−50	4
Cuneus		3.61	8	−76	28
Superior parietal lobule	1228	3.57	−2	−40	66
Primary somatosensory cortex		3.46	8	−44	68
Superior parietal lobule		3.44	−12	−40	52
Superior parietal lobule		3.41	−18	−42	42
Primary somatosensory cortex		3.29	0	−26	62
Superior parietal lobule		3.28	14	−48	72
Parahippocampal gyrus	314	3.73	26	−44	−8
Fusiform cortex		3.42	36	−46	−10
Parahippocampal gyrus		2.84	38	−26	−14
Fusiform cortex		2.58	38	−30	−16
Primary motor cortex	293	3.68	20	−18	66
Primary motor cortex		3.52	32	−28	62
Primary motor cortex		3.46	36	−30	66
Primary motor cortex		3.31	30	−28	68
Primary motor cortex		2.75	12	−28	72
Parahippocampal gyrus	258	3.51	−20	−42	−10
Fusiform cortex		3.43	−28	−48	−6
Heschl’s gyrus	226	3.48	54	−26	18
Secondary somatosensory cortex		3.36	52	−28	24
Heschl’s gyrus		2.85	62	−24	12
Secondary somatosensory cortex		2.73	40	−28	28
Supramarginal gyrus		2.61	48	−38	14
Angular gyrus		2.46	58	−36	18

**Table 3:** The group level activation results in (Craving>Neutral) contrast for MMT group.

**Anatomical Regions**	**Cluster Size**	**Z-Values**	**Local Maximum Co-Ordinates**		
Visual cortex	1426	3.8	20	−64	14
Lingual gyrus		3.6	0	−80	22
Lingual gyrus		3.56	2	−80	26
Cuneus		3.45	22	−64	2
Retrosplenial cortex		3.42	−10	−78	14
Cuneus		3.42	−16	−70	0
Insula	718	3.65	−48	−20	2
Inferior parietal lobule		3.57	−60	−38	20
Inferior parietal lobule		3.53	−50	−36	14
Inferior parietal lobule		3.43	−54	−34	12
Inferior parietal lobule		3.23	−60	−36	10
Insula		3.17	−38	−30	8
Primary somatosensory cortex	454	3.43	16	−46	56
Primary somatosensory cortex		3.4	18	−46	60
Superior parietal lobule		3.23	16	−46	52
Superior parietal lobule		3.23	24	−46	68
Superior parietal lobule		3.13	12	−50	68
Postcentral gyrus		3.06	12	−46	68
Secondary somatosensory cortex	263	3.44	50	−10	24
Secondary somatosensory cortex		3.25	58	−2	12
Primary somatosensory cortex		3.21	56	−6	22
Secondary somatosensory cortex		3.08	60	0	4
Secondary somatosensory cortex		2.87	58	−12	12
Secondary somatosensory cortex		2.85	64	−14	10
Heschl’s gyrus	262	3.3	56	−28	14
Inferior parietal lobule		3.19	56	−38	12
Inferior parietal lobule		3.18	58	−48	10
Heschl’s gyrus		3.12	60	−28	16
Superior temporal gyrus		2.96	60	−36	6
Inferior parietal lobule		2.91	46	−42	12

**Table 4:** The group level activation results in (Craving>Neutral) contrast for the control group.

**Anatomical Regions**	**Cluster Size**	**Z-Values**	**Local Maximum Co-Ordinates**		
Visual cortex	2214	3.97	−12	−58	0
Cuneus		3.94	−12	−46	−6
Visual cortex		3.87	−20	−58	2
Visual cortex		3.82	16	−62	8
Lingual gyrus		3.78	6	−64	2
Visual cortex		3.69	−8	−92	8
Inferior parietal lobule	705	4.19	−46	−34	26
Insula		3.51	−30	−36	12
Secondary somatosensory cortex		3.5	−40	−30	26
Insula		3.35	−34	−32	14
Insula		3.26	−48	−12	22
Primary somatosensory cortex		3.23	−16	−34	50
Supramarginal gyrus	649	3.98	50	−38	10
Middle temporal gyrus		3.51	54	−42	2
Inferior parietal lobule		3.45	58	−36	12
Insula		3.37	40	−24	10
Inferior parietal lobule		3.35	62	−32	18
Middle Temporal gyrus		3.24	64	−36	2
Cingulate gyrus	275	3.19	−10	−4	36
Primary motor cortex		2.71	−32	−16	36
Primary motor cortex		2.58	−26	−16	46
Inferior parietal lobule	230	3.82	−64	−44	14
Inferior parietal lobule		3.56	−48	−42	10
Inferior parietal lobule		3.09	−54	−46	22
Inferior parietal lobule		2.81	−62	−36	12

### 3.2. Dynamic causal modeling results

The time-series of each region was extracted according to the method introduced in the previous section. The DCM estimation process was done for each model in the model space and the resulting exceedance probabilities were used in the process of BMS algorithm. Family partitioning was done according to the nonlinear links and using BMA, the final DCM networks for all groups were calculated. The BMA results are presented in Table 5. Considering 4 connectivity matrices, this table is divided into 4 sections (highlighted with gray color).

**Table 5:** The BMA results. The links with zero strength in the table were not significant in their own groups. The last three columns of the table compare between groups with the P-values used in the test. In these columns “ns” means not significant.

	**Control**	**ABT**	**MMT**	**Significance**		

	**Mean Strength**	**Mean Strength**	**Mean Strength**	**Control-ABT**	**Control-MMT**	**ABT-MMT**
**Endogenous Connections**						
VMPFC to VMPFC	−0.1	−0.2	−0.1	ns	ns	ns
DLPFC to DLPFC	0	−0.8	0	P<0.01	ns	P<0.01
VS to VS	−0.1	−0.1	0	ns	P<0.05	P<0.05
AM to AM	0	−0.1	0	P<0.05	ns	P<0.05
VMPFC to DLPFC	0.2	0.2	0.1	ns	ns	ns
DLPFC to VMPFC	0.2	0.8	0.1	P<0.01	ns	P<0.01
VMPFC to VS	0.4	0.3	0.2	P<0.05	P<0.05	ns
VS to VMPFC	0.1	0.1	0.1	ns	ns	ns
VMPFC to AM	0.3	0.5	0.2	P<0.05	ns	P<0.05
AM to VMPFC	0.2	0	0.2	P<0.05	ns	P<0.05
DLPFC to VS	0.3	−0.4	0.1	P<0.01	P<0.01	P<0.01
VS to DLPFC	0	0.2	0.1	P<0.05	P<0.05	ns
DLPFC to AM	0.1	0.6	0.1	P<0.01	ns	P<0.01
AM to DLPFC	0.1	−0.2	0	P<0.01	P<0.05	P<0.01
VS to AM	0	0.2	0.1	P<0.05	P<0.05	ns
AM to VS	0.2	0.2	0.3	ns	ns	ns
**Craving Input**						
AM	0	0.7	0.1	P<0.01	P<0.05	P<0.05
**Craving Modulation**						
VMPFC to VS	0.1	0	0	P<0.05	P<0.05	ns
VS to VMPFC	0.1	0	0	P<0.05	P<0.05	ns
VMPFC to AM	0.2	0	0	P<0.05	P<0.05	ns
DLPFC to VS	−0.3	0	0	P<0.05	P<0.05	ns
DLPFC to AM	0.1	0	0	P<0.05	P<0.05	ns
AM to DLPFC	0.1	0	0	P<0.05	P<0.05	ns
**Nonlinear Connections**						
VMPFC to DLPFC-VS	0	−0.1	0.1	P<0.05	ns	P<0.01
VMPFC to VS-DLPFC	0	0.4	0	P<0.01	ns	P<0.01
VMPFC to VS-AM	0	0.2	0	ns	ns	ns
VMPFC to AM-VS	0	−0.2	0	P<0.01	ns	P<0.01
DLPFC to VS-VMPFC	−0.2	0	0	P<0.01	P<0.01	ns
VS to AM-DLPFC	−0.2	0	0	P<0.01	P<0.01	ns

**Figure 4:**
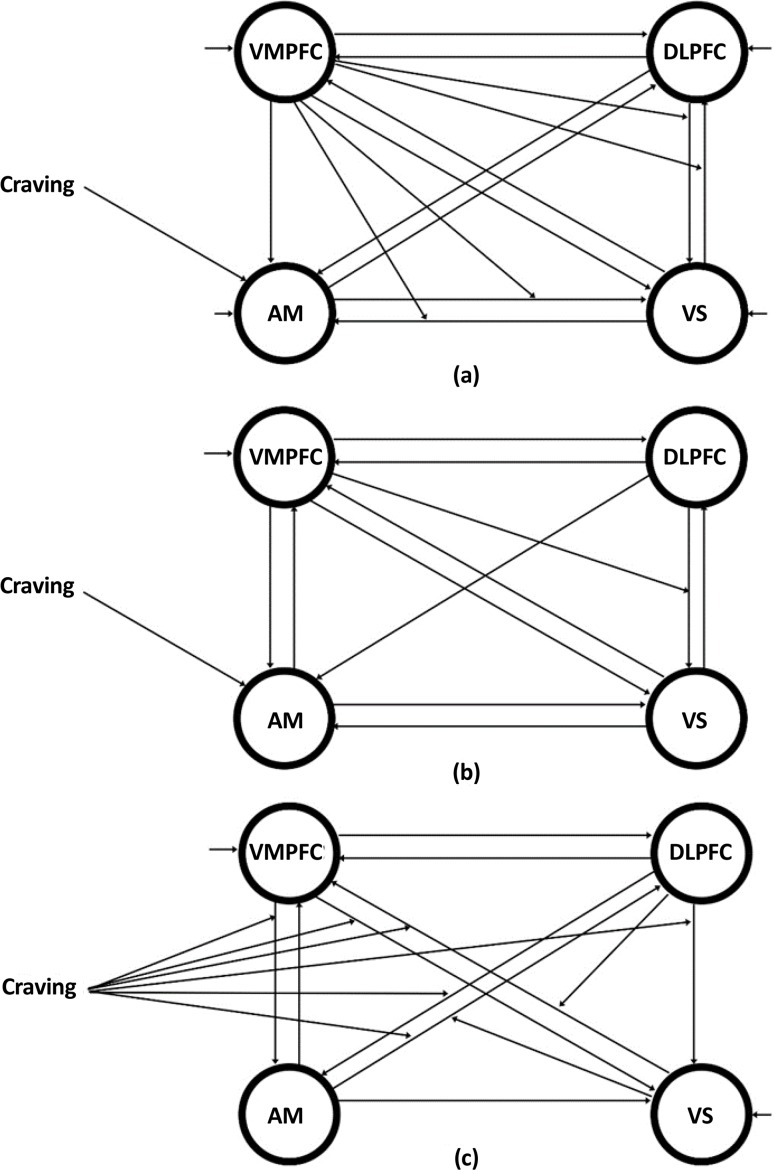
The DCM network structure for three groups; (a) the final network for ABT group, (b) The final network for MMT group, and (c) The final network for the control group.

The first section included the endogenous connections or the matrix A, the second section included the matrix B, the third section included matrix C, and the last included matrix D. The first column of the table presents the start and the end of each connection (for connections which do not exist in all groups, there are no rows). The next 3 columns are the mean strength of the named connection for each group. Zero number in the cells represents the lack of that connection in the relevant group. The numbers in these 3 columns represent different meanings with regard to effective connectivity theory; the change in the variance of the links starting point will change the variance of the links ending point by the factor of the links mean strength. The sign of the number is directly related to the correlation of the variance change in two signals; positive means directly correlated and negative means correlation with phase lag of 180°. The last 3 columns of the table present significant statistically meaningful differences between the groups (top of each column) in that link (the first column). These networks are depicted in Figure 4.

With regard to the effective connectivity network for the control group, the craving input link only modulated the links between the regions, but there was no modulation for the other two groups and the input link only affected the amygdala. In the control group, DLPFC and amygdala affected ventral striatum but were not influenced by it. In the ABT group, VMPFC affected amygdala but there was no reverse effect. Finally in the MMT group, the relation from DLPFC to amygdala was a 1-way connection. Self-inhibitory connections in the control group were limited to VMPFC and ventral striatum regions. In the MMT group, only VMPFC had this self-inhibitory effect, while in the ABT group, all regions had the self-inhibitory effect. In the control group, DLPFC affected the connection of ventral striatum to VMPFC, and ventral striatum itself affected the connection of amygdala to DLPFC. In the ABT group, VMPFC influenced reciprocal connections between DLPFC and ventral striatum and also the connections between ventral striatum and amygdala. In the MMT group, the nonlinear link was from VMPFC to the link from DLPFC to ventral striatum.

## 4. Discussion

There are studies addressing the results of various treatment methods on drug abusers, but there is no conclusive evidence for superiority of any treatment method over others (Wang, et al., 2011). In addition, studies have shown different brain activation patterns for treated subjects, however, currently there is no direct study of brain effective connectivity differences between variously treated subjects. The effective connectivity estimation can measure the regulatory effects of regions and craving cues on the different parts of the brain network and helps in better understanding of the craving mechanism (Kober, et al., 2010).

We investigated the effective connectivity network in 3 groups. Two groups included successfully treated drug abusers, with different treatment methods; one with MMT and the other with ABT. The third group included individuals with no history of drug abuse. The results of the last group helped us provide a basic network for brain effective connectivity pattern, when no prior bias exist. The networks were estimated between 4 regions of interest which were mentioned in previous studies. Studies have indicated that cue exposure increases craving and results in more activities in these regions. These regions are also associated with emotion.

The implication of prefrontal regions in cognitive control (Wrase, et al., 2007; de Greck, et al., 2009) and its regulatory effect on emotion specific regions like amygdala and ventral striatum have been already proven (Franklin, et al., 2007; Meda et al., 2009). The resulted networks showed the active role of these regions in the network and by using DCM, the difference of the networks between these 3 groups were depicted numerically and structurally.

### 4.1. The craving input effect

The differences between the control group and two treated groups indicated that the cue-induced pictures did not affect the emotion of healthy subjects, but affected amygdala, causing emotional indices in the other groups. To the healthy subjects, these pictures seem as neutral pictures and only modulate some of the links in the network (as perturbations for the task). This finding is in accordance with previous studies which mentioned the role of amygdala in cue-induced tasks in subjects with a history of drug abuse (Bechara, et al., 1999; Bechara, et al., 2003; Goudriaan, et al., 2010; Chase, et al., 2011; Tang et al., 2012; Ray, et al., 2015).

### 4.2. The modulatory effect of ventromedial pre-frontal cortex

Studies have already proven the role of prefrontal-striatal pathway in the control of craving to drug use (Koob, 2001; Volkow, Fowler, Wang, 2003; Everitt & Robbins, 2005). The lack of control over drug taking is considered a sign of addiction and is critical in relapse. Frontal brain regions have an important role in inhibitory control of this behavior. It has been shown that VMPFC has modulatory effects on other regions, including amygdale and ventral striatum in the cue-induced craving tasks (Bechara, et al., 1999; Bechara 2005; Lu, et al., 2012; Sladky, et al., 2013). In our study by considering the modulatory effect of VMPFC on other interregional connections, it was revealed that in healthy subjects there was no modulatory effects, as they did not have any emotional responses to craving specific pictures.

However, the VMPFC modulatory effects in ABT group indicate the important role of this region in drug resistance among these subjects. This group are trained to resist drug taking and the results reveal that the VMPFC acts as a part of top neural system, which tries to control the emotional decisions. This finding is in accordance with the findings of prior studies on the regulation of negative emotions (Ochsner & Gross, 2008) and positive emotions (Kim and Hamann, 2007; Delgado, Gillis, Phelps, 2008). They reported the activation of these prefrontal systems and suggested the role of cognition to regulate responses to affective cues; for example drug cues. In the MMT group, we did not expect to observe any modulatory effect as the subjects took a dose of methadone and they would seem to have normal behavior when encountering cue-induced images. The only modulatory effect of VMPFC in this group was observed in the DLPFC-ventral striatum link, which was not statistically different from the control group who did not have this link (in the control group this link was not statistically significant).

The importance of DLPFC as a part of prefrontal cortical area has been highlighted in previous addictive-cue studies (Goldstein & Volkow, 2002; Wilson et al., 2004; Goldstein, et al., 2007; Volkow, Fowler, Wang, Baler, & Telang, 2009). The neural activity of DLPFC may be modulated by sensory information, motivational state, and task contingencies (Miller, 2000). In our study, DLPFC exhibited an important role in regulatory processing of cognitive and motivationally relevant information.

In this study, we identified 3 effective connectivity networks for 3 different groups, with various structures and links strength. These results can prove the different effects of treatment methods and their underlying neuronal mechanisms. The vast modulatory effect of VMPFC occur only in ABT group. This effect can prove the inhibitory role of this brain region in drug craving and also indicate the successful treatment of the subjects in this group, as the main target of this treatment is to train the patients to control their craving. The modulatory effect of VMPFC in MMT group, in contrast with the other treatment group, is a single effect which can exist because of the anatomical connections between these regions.

In the control group, the input data did not directly affect the regions and only modulates the links. In this network, input is on when the craving images are shown and is off in other situations. Thus, the input can be interpreted as watching craving-related images and this exclusive modulatory effect can be the result of subjects’ inattentiveness to these cues. These results cannot prove the superiority of one method over another but at least may help choose the best method for different subjects in various situations.
